# Dimerization: a structural feature for the protection of hepatitis E virus capsid protein against trypsinization

**DOI:** 10.1038/s41598-018-20137-2

**Published:** 2018-01-29

**Authors:** Wenjuan Wei, Nouredine Behloul, Sarra Baha, Zhenzhen Liu, Mehwish Saba Aslam, Jihong Meng

**Affiliations:** Department of Microbiology and Immunology, School of Medicine, Southeast University, Nanjing, Jiangsu province China

## Abstract

Orally-transmitted viruses have evolved in a way to resist the extreme conditions of the host’s gastrointestinal environment, especially the proteolysis of their structural proteins. However, the mechanisms allowing these viruses to survive these harsh conditions remain unclear. Hepatitis E virus (HEV) is an orally-transmitted human pathogen. Its capsid protein contains three domains S, P1 and P2. The latter forms a homodimer protruding from the virus shell, making it the most exposed part. By combining biochemical and computational methods, we found the trypsin digestion sites to be highly conserved among the HEV strains. Furthermore, the constructs of the HEV capsid protein that contain an extended P2 domain were digested within the extensions leaving the P2 domain intact. The trypsinization seems to occur in three possible double cleavages at R451-R619, R460-R619 or R460-R631.The dimerization disrupts the trypsin action at three main sites in the P2 domain R542, K544 and K554. These sites are very exposed in the monomeric P2 domain constructs which makes the monomeric forms very susceptible to trypsin action. Therefore, we believe that dimerization is a structural feature that has been selected by the evolutionary forces to render the HEV capsid protein resistant to the host’s proteases; an evolutionary feature that could be common to some other (if not all) orally-transmitted viruses.

## Introduction

Hepatitis E virus (HEV) is a non-enveloped virus with a positive-sense, single-stranded RNA genome (7.2 kb) which belongs to the Orthohepevirus genus in the family of the Hepeviridae. It causes both acute hepatitis outbreaks in developing countries and sporadic cases worldwide^1^. HEV infection usually results in a self-limited, acute illness. Although most patients recover, the mortality among pregnant women is often 10–25%^2^; it can also become chronic in immuno-compromised patients such as organ-transplant recipients and HIV-infected patients^3^; and liver fibrosis can progress rapidly in HEV infected patients leading to cirrhosis in 2–3 years^4^. Therefore, HEV infection is a major health concern worldwide.

There are currently four described genotypes that infect humans (genotypes 1, 2, 3 and 4). Genotypes 1 and 2 only infect humans, whereas genotype 3 and 4 are zoonotic, present in different animals, particularly feral and domestic pigs^5^. The HEV genome consists of 3 open reading frames (ORFs). ORF2 encodes a single structural protein (pORF2) of 660 amino acids (aa), composed of three domains (S、P1 or M and P2 or P)^6,7^. The P2 domain, also referred to as the E2s domain, forms an intimate homodimer in solution^8^. Currently, two recombinant vaccines based on human-HEV sequences have undergone clinical trials in China. The first commercial hepatitis E vaccine (Hecolin) has been licensed in China in 2012. It was derived from HEV genotype 1 (aa 368–606 of ORF2) and produced in bacterial cells^9^. Another HEV virus-like particle (VLP) vaccine named p179 (aa 439–617 of ORF2 protein), derived from HEV genotype 4 and expressed in *Escherichia coli* (*E. coli*), was developed by our laboratory and the Changchun Institute of Biological Products^10^.

The HEV capsid is formed by capsomeres consisting of homodimers of a single structural capsid protein. These dimers are believed to protrude from the viral surface and to interact with host cells to initiate infection. A study of E2 protein (aa 394–606) suggested that the dimeric domain encompasses aa 459–606, of which, aa 597–602 were involved in the dimer formation^11^. The HEV dominant neutralizing epitopes were located within the P2 domain (aa 455–602)^12,13^. Li, *et al*. reported that the HEV dominant neutralizing epitopes were located within the E2s domain (aa 455–602) and the recognition of these sites by the neutralizing antibodies was dependent on the dimeric state^8^. However, we previously expressed, for the first time, in *Pichia pastoris* (*P. pastoris*) single-point mutated p179 proteins (aa 439–617, mutated at N562) that were naturally expressed as monomers and both were reactive against the anti-HEV neutralizing antibodies, indicating that the dimerization was inessential for p179 proteins interaction with the neutralizing antibodies^14^.

HEV is enterically-transmitted, which implicates that HEV capsid protein must be able to resist the proteolytic degradation in the gastrointestinal (GI) tract. In order to gain insights into the proteolysis resistance of HEV capsid protein, we adopted computational and biochemical methods to investigate the resistance of different ORF2 proteins to trypsin proteolysis, since this latter is one of the major GI tract proteases. Further, we expressed the mutated p179 proteins, naturally occurring as monomers, in *E. coli* and investigated the influence of protein structure and dimerization state on the proteolytic resistance of HEV capsid proteins.

## Results

### Expression and purification of truncated HEVORF2 proteins

**Figure 1:**
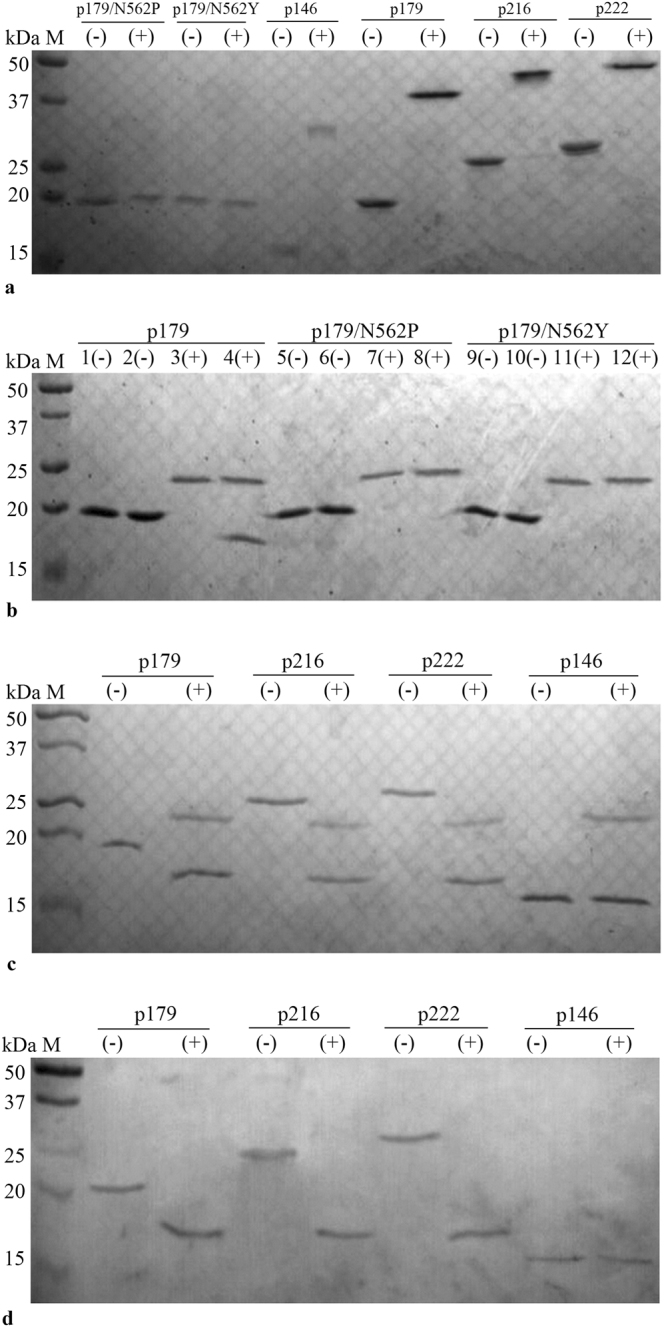
(**a**) Non-reducing SDS-PAGE gel of p179/N562P, p179/N562Y, p146, p179, p216 and p222. (−) unheated, (+) heated. Full-length gel is presented in Supplementary Figure S3. **(b)** SDS-PAGE gel of the wild-type and mutant p179/N562 after trypsin-proteolysis: (+) treated with trypsin; (−) not treated with trypsin. Lanes 1, 3, 5, 7, 9, 11: heat-denatured before trypsin treatment and before loading onto the gel; Lanes 2, 4, 6, 8, 10, 12: heated-denatured only before loading onto the gel without trypsin treatment. M: protein marker in kilo-Daltons (Bio-Red, USA); Trypsin molecular weight is about 24 kDa. Full-length gel is presented in Supplementary Figure S4. (**c**) SDS-PAGE gel of HEV ORF2 proteins after trypsin-proteolysis. Full-length gel is presented in Supplementary Figure S5. (**d**) Immunoreactivity of HEV ORF2 proteins with mAb 1G10 detected by Western blot. (+): treated with trypsin; (−): not treated with trypsin. All samples were boiled only before loading on the gel. Full-length gel is presented in Supplementary Figure S6. M: protein marker in kilo-Daltons (Bio-Red, USA). Trypsin molecular weight is about 24 kDa. The grouping of gels/blots are cropped from different gels and made explicit using dividing lines.

The different coding sequences were amplified from HEV ORF2, purified and then separately inserted into pET-28a (+) vector. The different constructs were used to transform *E. coli* (BL21) competent cells. Restriction digestion and DNA sequence analysis confirmed that the coding sequences were inserted correctly without any shifting or mutations. Next, the proteins were expressed successfully and the size of each recombinant protein was in agreement with the expected molecular weight: P146 (16.86 kDa), p179 (20.27 kDa), 216 (24.35 kDa) and 222 (25.16 kDa). All the expressed proteins were soluble, and purified using Ni–NTA affinity chromatography under native conditions. All these 3 proteins were confirmed to dimerize in a solvent environment by using a non-reducing sodium dodecyl sulfate polyacrylamide gel electrophoresis (Non-reducing SDS-PAGE) as shown in Fig. 1a.

### Characterization of wild-type and mutant p179N562

Two p179 mutant clones containing substitutions p179N562P and p179N562Y were constructed as described in the Methods section, and were successfully expressed in *E. coli*. SDS-PAGE analysis of the wild-type and mutated proteins revealed that after heat treatment, the three proteins had a molecular weight of approximately 20 kDa, which corresponds to the molecular weight of the p179 monomer. However, without heat treatment, the wild-type p179 band was observed at 40 kDa corresponding to the molecular weight of the p179 homodimer; while the bands of the two mutant proteins were both appeared at 20 kDa (Fig. 1a). These results indicated that the wild-type p179 protein formed homodimers, whereas the two mutants could not dimerize. These observations are consistent with the previous results obtained using a eukaryotic expression system^14^.

### Evaluation of trypsin-resistance in wild-type and mutant p179 proteins

The results are shown in Fig. 1b. After an incubation period of 2 hours at 37 °C with trypsin, there was no visible band in the heat denatured wild-type p179 lane on the SDS-PAGE, indicating that it was completely digested by trypsin. Likewise, the same results were observed with the non-denatured p179 mutants. By contrast, non-denatured wild-type p179 was cut into a 17–17.5 kDa fragment by trypsin. The heat treatment reduced the wild-type p179 dimers into monomers, and the two mutant proteins were naturally occurring as monomers. Therefore, this assay revealed that the trypsin digestion of the monomers was complete, while in the dimers, the proteolysis led to a lighter fragment. This indicated that the dimerization of the p179 protein played a role in preventing the complete degradation by trypsin.

### Trypsin action on the truncated HEV ORF2 proteins of different lengths

To further investigate the action of trypsin on HEV ORF2 proteins, we subjected p146, p179, p216 and p222 proteins to highly proteolytic milieu containing trypsin as described in the Methods section.

SDS-PAGE analysis showed that the molecular weight of the p146 monomer, did not changed after trypsin treatment, and the band appeared around 16–17 kDa. Interestingly, after tryptic digestion of p179, p216 and p222, three respective bands were observed around 17–17.5 kDa (Fig. 1c), indicating that the yielded products could be the same fragment since these three protein sequences were overlapping and all of them comprise the P2 domain: p179 (aa 439–617), p216 (aa 422–637) and p222 (aa 439–660).

Furthermore, Western blot analysis showed that all the truncated proteins (p146, p179, p216 and p222) and the proteolysis products were strongly reactive against the HEV-neutralizing 1G10 monoclonal antibody (Fig. 1d). This indicated that the trypsin action did not affect the exposure of the neutralizing epitopes.

### Trypsin and HEV ORF2 proteins docking and prediction of the probable trypsin proteolytic sites

**Figure 2:**
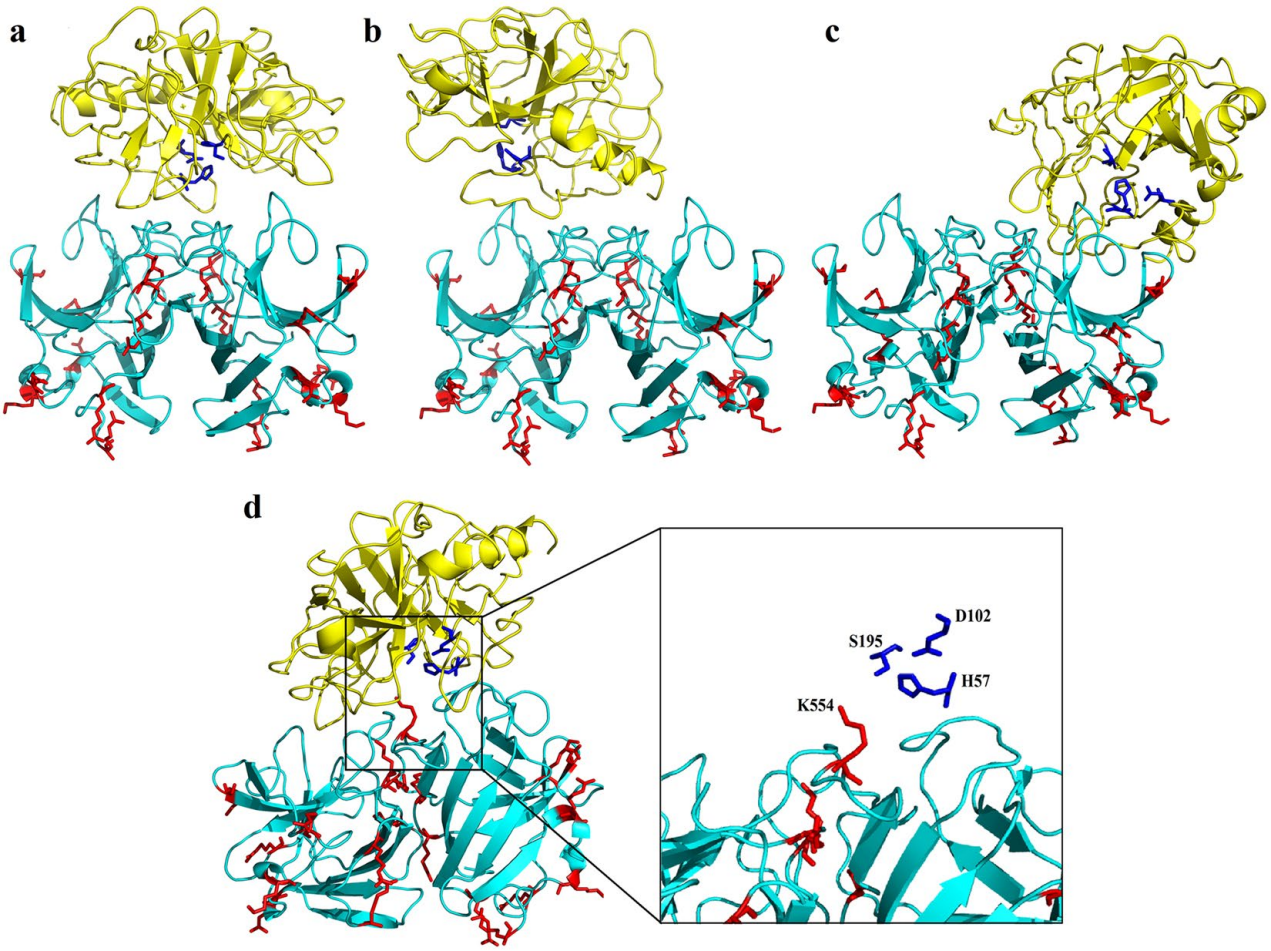
Illustration of the best docking poses (1–4) of trypsin (PDB ID: 418 G) and p146 homodimers. The trypsin and the p146 homodimer are shown in yellow and cyan cartoon representation, respectively. The trypsin catalytic triad is represented in blue sticks and the trypsin digestion sites depicted in red.

**Figure 3:**
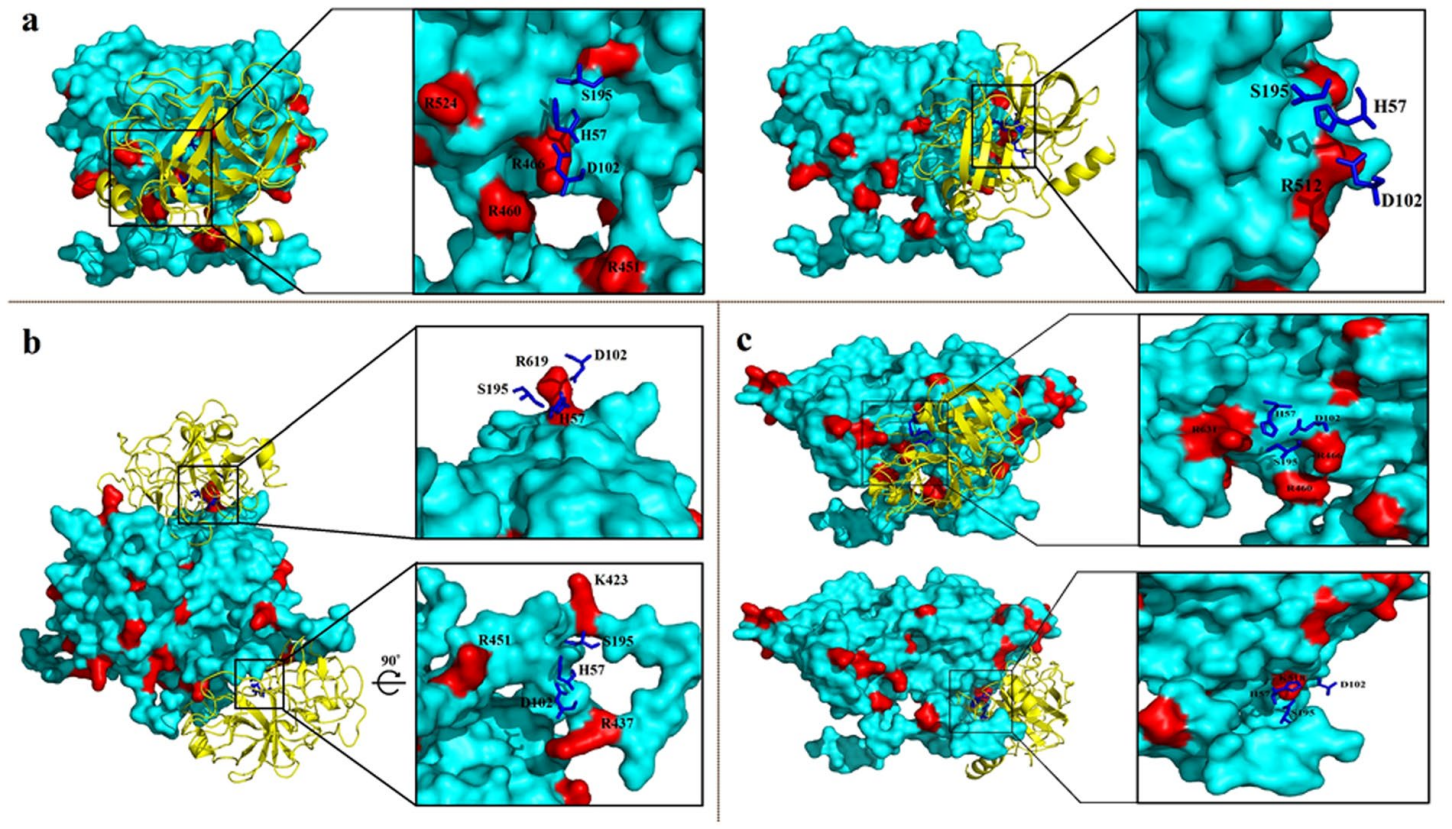
Illustration of the best docking poses of trypsin (PDB ID: 418 G) and p179 (**a**), p216 (**b**) and p222 (**c**) homodimers. The trypsin is shown in yellow cartoon and its catalytic triad blue sticks. p179, p216 and p222 homodimers are shown in cyan surface representation with the trypsin digestion sites depicted in red.

The 3D structure models of all the proteins were predicted using Phyre2 protein fold recognition server. After refinement and quality assessment, only the best ranked models were selected for further analysis. The protein–protein docking was performed using Z-Dock, a total of 2000 poses were generated^15^. The top 500 docking poses, as scored by Z-Rank, were chosen for an R-Dock refinement^16^. Next, the refined poses were visualized using Pymol. All the dockings where the catalytic site of trypsin^17^ and the proteolytic sites (R or K) of the HEV proteins were not within the docking interface, were ruled out. The docking poses that engaged the catalytic site and one or more R/K residues are shown in Figs 2,3 and S1.

Using the proteins dimers for docking revealed that for p146, only K554 met the requirements (Fig. 2). However, cutting the p146 protein at this site would lead to the formation of a smaller fragment or to the total degradation, and neither of these possibilities has been observed on the SDS-PAGE after trypsin treatment. For p179, five residues in the N-terminus were found to be possible proteolytic sites R451, R460, R466, R512 and R524 (Fig. 3a). Four residues in p216 (K423, R437, R451and R619) and four others in p222 (R460, R466, K518 and R631) were found close to trypsin catalytic sites (Fig. 3b and c respectively). The above potential proteolytic sites and the resulting fragments after trypsin digestion are summarized in Table 1 (see also Figure S2). These results indicated that for p179, only when the proteolysis occurs in three sites (R451, R460 or R466) would lead to formation of fragments of molecular weights (17~19 kDa) similar to those observed in the trypsin digestion assay. For p216, only double cuts at R437-R619 (19.85 kDa) and R451-R619 (18.10 kDa) would be in agreement with the results of the digestion assays. Likewise, only two double cuts were probable for p222; the first at R460-R631 (18.64 kDa) and the second at R466-R631 (17.94 kDa).

**Table 1:** Summary of possible trypsin proteolytic sites and comparison of the predictions with trypsin digestion assay results.

Proteins	Proteolytic sites^a^	Longest resulting peptide (aa)	Molecular weight (kDa)	Result of trypsin digestion assay
**146**	**FL**	**153**	**16.68**	**16.5–17**
	554	100	11.01	
179	**FL**	**185**	**20.27**	**17–17.5**
	451	172	18.62	probable^b^
	460	163	17.73	probable
	466	157	17.03	probable
	512	111	12.15	
	524	99	10.79	
**216**	**FL**	**222**	**24.35**	**17–17.5**
	423	220	24.10	
	437	206	22.63	
	451	192	20.89	
	619	198	21.56	
	423–619^c^	196	21.32	
	437–619	182	19.85	probable
	451–619	168	18.10	probable
**222**	**FL**	**228**	**25.16**	**17–17.5**
	460	206	22.62	
	466	200	21.93	
	**518**	148	16.41	
	631	193	21.17	
	460–631	171	18.64	probable
	466–631	165	17.94	probable
	518–631	113	12.29	

FL: Full length protein; ^a^All the proteolytic sites were predicted via protein docking analysis; ^b^The molecular weight of longest resulting peptide is in agreement with the results of proteolytic digestion assay; ^c^the protein was digested at two sites at the same time.

All the possible cuts in the region aa 422–660 of the ORF2 protein and the molecular weight of the resulting peptides were exhaustively summarized in figure S2.

### The solvent accessibility and exposure of lys/arg (K/R) residues

The degree of amino acid exposure to solvent is important for residue hydrolysis and the residue solvent accessibility is a key factor that affects the hydrolysis ability of trypsin. Therefore, we sought to determine the degree of exposure and solvent accessibility of all the cleavage sites within the HEV ORF2 proteins investigated in this study. The results are shown in Table 2.

**Table 2:** The residue accessible surface area (RSA), percent solvent accessibility (PSA) and protrusion index (PI) of trypsin proteolytic sites.

Sites	p146			p179			p216			p222		
	RSA (Å^2^)	PSA (%)	PI	RSA (Å^2^)	PSA (%)	PI	RSA (Å^2^)	PSA (%)	PI	RSA (Å^2^)	PSA (%)	PI
**K423**	—	—	—	—	—	—	**176.5**	**90.8**	**0.9**	—	—	—
**R437**	—	—	—	—	—	—	**227.2**	**99.1**	**0.9**	—	—	—
**R451**	—	—	—	**184.0**	**80.2**	**0.9**	**156.2**	**68.1**	**0.7**	**137.9**	**60.1**	**0.9**
R460	109.3	47.7	0.8	142.2	62.0	0.5	43.6	19.0	0.4	130.5	56.9	0.4
**R466**	**123.7**	**53.9**	**0.8**	159.4	69.5	0.2	88.7	38.7	0.1	159.5	69.5	0.2
**R512**	**132.4**	**57.7**	**0.6**	**135.3**	**59.0**	**0.7**	**154.4**	**67.3**	**0.7**	96.9	42.2	0.5
**K518**	**140.9**	**72.5**	**0.9**	**148.1**	**76.2**	**0.7**	**157.5**	**81.0**	**0.7**	146.9	75.5	0.3
R524	111.9	48.8	0.9	**159.9**	**69.7**	**0.8**	118.2	51.5	0.4	44.9	19.6	0.3
K534	44.7	23.0	0.6	61.1	31.4	0.6	95.9	49.3	0.5	46.4	23.8	0.3
R542	21.0	9.1	0.6	77.4	33.7	0.3	50.1	21.9	0.0	41.8	18.2	0.4
K544	19.4	10.0	0.4	0.0	0.0	0.3	1.5	0.8	0.1	25.9	13.3	0.5
K554	89.6	46.1	0.9	23.0	11.8	0.9	5.5	2.8	0.4	89.6	46.1	0.8
R578	76.6	33.4	0.3	91.0	39.7	0.4	90.9	39.6	0.3	18.1	7.9	0.2
**R619**	—	—	—	—	—	—	**216.6**	**94.4**	**0.9**	**193.6**	**84.4**	**0.9**
**R631**	—	—	—	—	—	—	43.9	19.1	0.1	**161.2**	**70.3**	**0.6**
**R649**	—	—	—	—	—	—	—	—	—	**146.9**	**64.1**	**0.8**
**K651**	—	—	—	—	—	—	—	—	—	**139.5**	**71.7**	**0.8**
**K653**										**147.3**	**75.8**	**0.9**
K656	—	—	—	—	—	—	—	—	—	76.9	39.5	0.6
R658	—	—	—	—	—	—	—	—	—	93.6	40.8	0.7

Trypsin cleavage sites with high solvent accessibility are in bold.

A total of 20 proteolytic sites have been found within the portion of HEV ORF2 protein covered by the different proteins investigated in this study (aa 422–660). It has been previously reported that the ORF2 protein can be divided into three separate domains: domain S (aa 118–313); domain P1 (aa 314–453) and domain P2 (aa 454–606)^7^. Accordingly, among the 20 possible proteolytic sites, three are located within the P1 domain (K423, R437 and R451); ten are located within the P2 domain (R460, R466, R512, K518, R524, K534, R542, K544, K554 and R578) and seven within the C-terminal end (R619, R631, R649, K651, K653, K656 and R658). The three sites located within the P1 domain showed high solvent accessibility and high protrusion index in all the proteins containing this fragment; likewise, five out of the seven sites in the C-terminal end were also very exposed (R619, R631, R649, K651 and K653). Concerning the sites within the P2 domain, none was exposed in p222 protein, while two residues, namely R512 and R518, were exposed in the three other proteins.

By combining the bioinformatics analyses and the experimental results, it appeared that the p146 was not digested at K554 because the predicted molecular weight of resulting peptide was inconsistent with the experimental results and the exposure of this site was very low. For the other three proteins the proteolysis has very probably occurred at the beginning of the P1 domain portion (at aa 451 in p179 and /or aa 460/466 in p216 and p222) and within the C-terminal end at aa 619 for both p216 and p222.

### Computational analysis of dimeric and monomeric proteins

**Figure 4:**
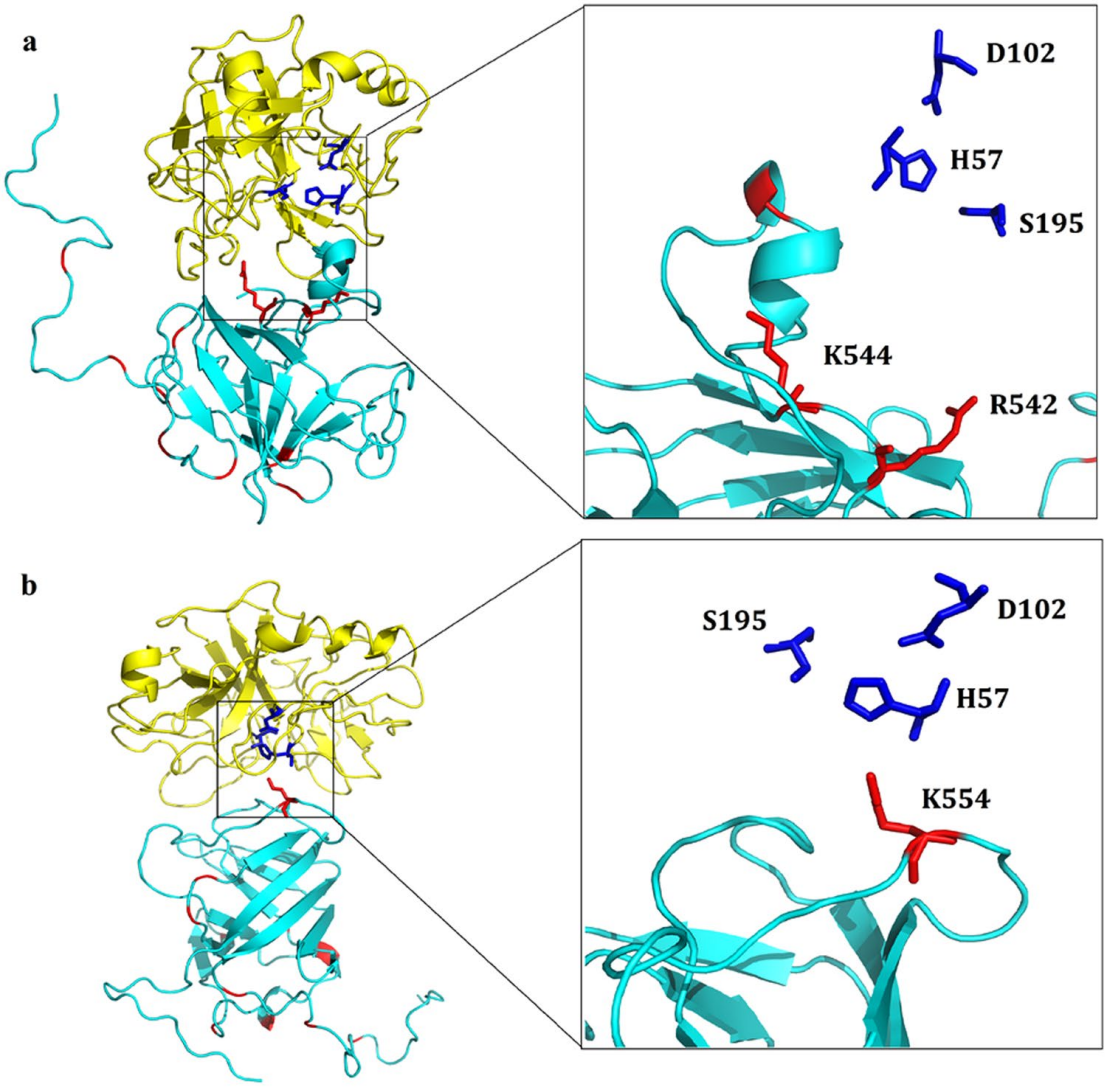
Illustration of the docking results of p179 monomer and trypsin (PDB ID: 418 G). The trypsin and the monomeric p179 are shown in yellow and cyan cartoon representation, respectively. The trypsin catalytic triad is shown in blue sticks and the proteolytic sites in p179 (K554, K544 and R542) in red sticks.

The trypsin digestion assay revealed that the p179 monomers were completely digested while the dimerized proteins yielded a smaller fragment of about 17–17.5 kDa. Therefore, we performed another protein docking to analyze the interaction between monomeric HEV ORF2 proteins and trypsin. Besides the sites identified by using the dimerized proteins (see sections above and Figs 2 and 3), three other residues have been identified as potential cleavage sites R542, K544 and K554 (Fig. 4).

Furthermore, solvent accessibility calculations revealed that these three sites were more exposed in the monomer than in the dimeric form, while all the other K/R sites have exhibited the same solvent accessibility in both forms as shown in Table 3. Taken together, these results indicated that the exposure of these particular sites makes a plausible explanation for the total digestion of the monomers but not the homodimers.

**Table 3:** The difference of percent solvent accessibility (%) of trypsin digestion sites between dimeric and monomeric HEV ORF2 proteins.

Sites	p179		p216		p222	
	Monomer	Dimer	Monomer	Dimer	Monomer	Dimer
K423	—	—	90.783	90.783	—	—
R437	—	—	98.394	99.052	—	—
R451	80.214	80.214	67.897	68.116	60.118	60.118
R460	62.004	62.004	18.996	18.996	57.106	56.886
R466	69.502	69.502	38.658	38.658	69.311	69.523
R512	59.636	58.978	67.519	67.307	43.937	42.238
K518	76.155	76.155	81.21	80.967	75.522	75.522
R524	69.927	69.708	51.539	51.539	19.562	19.562
K534	31.437	31.437	49.311	49.311	23.842	23.842
**R542**	**65.977**	**33.734**	**48.992**	**21.862**	**40.714**	**18.203**
**K544**	**9.126**	**0.00**	**44.849**	**0.751**	**66.505**	**13.34**
**K554**	**71.144**	**11.803**	**23.692**	**2.805**	**46.055**	**46.055**
R578	39.671	39.671	39.643	39.643	8.294	7.869
R619	—	—	94.439	94.439	84.613	84.394
R631	—	—	19.139	19.139	70.261	70.261
R649	—	—	—	—	63.832	64.052
K651	—	—	—	—	71.739	71.739
K653	—	—	—	—	76.787	75.769
K656	—	—	—	—	39.522	39.522
R658	—	—	—	—	40.799	40.799

### Conservation of the K/R sites among different HEV strains

The 20 K/R residues investigated in this study were found to be highly conserved among the HEV strains with the conservation rate of 97.8 to 100% as listed in table S1. It is worth noting that most of the mutations were KΔR substitutions, which makes the rate of conservation even higher in terms of trypsin proteolytic sites. Substitutions by other amino acids were all found in strains isolated from animals: 451RΔQ in a strain from rabbits; 524RΔQ in strains from camels; 619RΔC and 649 RΔP in strains from swine; and 653 RΔE in wild boar and laboratory strains. It is also to mention that except for camel and rabbit strains, all mutations to other than K/R residues are located within the C-terminal end out of P2 domain.

## Discussion

Virulence characteristics of enterically-transmitted viruses, such as HEV, hepatitis A virus and norovirus, enable them to initiate infection, spread in the body, and replicate to large copy numbers that leads to the impairment of the target cells^18^. To maintain their pathogenicity, the first crucial step for these viruses is to survive the extreme conditions of the gastrointestinal environment especially the proteolysis degradation of their structural proteins.

Therefore, in the present study, we sought to gain insights into how these viruses might escape the gastrointestinal proteolysis, by investigating the susceptibility of truncated HEV ORF2 proteins to trypsin digestion. Herein, we report for the first time that the HEV ORF2 proteins are found to be resistant to trypsinization, and the dimerization of these proteins plays an important role in protecting the HEV capsid from being destroyed.

The HEV capsid protein (660 aa) contains different domains as reported by Guu *et al*.^7^: domain S comprises the region 118–313 aa and forms the viral shell; domain P1 comprises the region 314–453 aa and forms a surface plateau at 3-fold-related axes of the virus capsid; domain P2, at position 454–606 aa; and a 607–660 aa fragment referred to, in this study, as the C-terminal end. Domain P2 forms a protruding spike from the shell, is responsible for cell-attachment and contains the dominant neutralizing epitopes^9,12,19,20^ making it thus the most exposed region to the gastrointestinal juice.

Therefore, we expressed 4 HEV ORF2 proteins of different lengths that all contain the P2 domain, covering all together the region aa 422–660: p146 (aa 460–605), p179 (aa 439–617), p216 (aa 422–637) and p222 (aa 439–660). Subsequently, these four proteins were confirmed to form stable homodimers in natural conditions, which is consistent with the previously expressed HEV ORF2 proteins^14,21,22^. We also expressed in *E. coli* two mutated p179 proteins namely p179/N562P and p179/N562Y that were previously produced in a eukaryotic system^14^. The N562 residue plays a key role in forming and maintaining the p179 homodimers and its mutation led to the expression of fully functional p179 mutated proteins that can no longer dimerize in natural environment, which is very suitable for the study of the effects of ORF2 protein dimerization on different aspects of the HEV life cycle.

In the tryptic digestion assay, the wild-type p179 was digested into a smaller fragment of about 17~17.5 kDa while the mutants were completely degraded. Reducing the wild-type p179 into monomers by heat treatment prior to trypsinization led to its full degradation. This indicates that the heat treatment altered not only the quaternary structure but also the tertiary structure as it has been discussed previously^8,14^ where the heat denaturation disrupted the reactivity of the ORF2 proteins against the neutralizing monoclonal antibodies. Therefore, we concluded that dimerization might play a crucial role in the resistance of the wild-type p179 against full trypsin degradation.

To further confirm these conclusions, we adopted two computational approaches: the protein-protein docking and the analysis of the cleavage sites exposure. Trypsin catalytic triad consists of His-57, Asp-102, and Ser-195^17^. These three residues form a charge relay that increases nucleophilicity of the active site (Serine). Therefore, in the first approach two conditions were taken into account during the selection of the best docking solutions: (1) the docking interface must engage the trypsin catalytic triad and one or more cleavage sites on the ORF2 proteins; (2) The pose score must be high, indicating that theoretically the docking solution would occur spontaneously in a solvent environment. Accordingly, we have identified the highly probable digestion sites by analyzing all the possible trypsinization products. For p179, only when the proteolysis occurs in three sites (R451, R460 or R466) would lead to formation of fragments of molecular weights (17~19 kDa) similar to those observed in the trypsin digestion assay. For p216, only double cuts at R437-R619 (19.85 kDa) and R451-R619 (18.10 kDa) would be in agreement with the results of the digestion assays. Likewise, only two double cuts were probable for p222; the first at R460-R631 (18.64 kDa) and the second at R466-R631 (17.94 kDa). This was further confirmed by the second approach calculations that showed all the above-mentioned residues to be highly exposed compared to the other sites. On the other hand, we have previously reported the use of the neutralizing 1G10 mAb for mapping the dominant neutralizations epitopes within the HEV capsid protein^13^. The results indicated that only the truncated ORF2 proteins containing the region aa 477–613 could react with the 1G10 mAb, suggesting that the neutralization epitope(s) of HEV genotype 4 is located between aa 477 and aa 613. More specifically, fragments shorter by one amino acids in either ends could no longer react against the 1G10 mAb (aa 478–613 and aa 477–612). It is worth mentioning that the HEV strain used in this previous study has an insertion of 12 amino acids in the capsid protein^23,24^ and by aligning it with the sequence used in the present investigation, we found that the aa 477 and aa 613 correspond to aa 465 and aa 601, respectively. Herein, all the trypsinization products reacted against the 1G10 mAb, indicating that they all comprise the region aa 465–601 of the P2 domain, thus rolling out the R466 from the list of probable cleavage sites. Therefore, these observations taken all together (summarized in figure S2), we concluded that the tertiary structure and the antigenic composition of the P2 domain were conserved in the trypsinization products and the digestion occurred in only three possible ways within the P1-P2 connecting region and within the C-terminal end extensions: R451-R619, R460-R619, and R460-R631.

Then, we applied the same computational methods to elucidate how the dimerization could protect the P2 domain from trypsinization. Indeed, besides the proteolytic sites identified above, three other sites (R542, K544 and K554) were found to be more exposed in the monomers and very accessible to the catalytic triad of trypsin. This could therefore explain why the monomers were more susceptible to trypsin digestion and highlight the role of dimerization in P2 domain resistance to trypsin.

Structural studies enhanced our understanding of the general architecture of the HEV capsid and the mechanism underlying its assembly^7,25^. The P1 and P2 domains are connected by a long flexible linker (^445^NQHEQDRPTPSPAPSRPF^462^) that allows a proper dimerization of the P2 domain^7^. Two of the trypsin digestion sites are located within this flexible hinge R451 and R460. As noted by Guu *et al*.^7^, this region is rich in proline and thus it is a poor substrate for proteases in general and for trypsin in particular^26^. Given the transmission route of HEV, the evolutionary forces may have selected sequence and structural features (proline-rich linker and P2 domain dimerization, respectively) that make the HEV capsid protein highly resistant to trypsin but more likely, for all the other gastrointestinal proteases. However, further investigation is needed to establish the relationship between these features and the protease resistance of the fecal-orally transmitted viruses.

The 20 trypsin digestion sites studied in the present work are highly conserved among the HEV strains, irrespective of genotype or host. None of these residues was reported to participate in forming or maintaining the dimer architecture^8^. Therefore this relative conservation being retained under selection pressures suggests that it might be essential for virus survival and replication *in vivo*, especially virus-host first interaction as discussed previously by Li *et al*. concerning R512^8^, R578 and K554^9^. We can further speculate that the dimerization of the P2 domain could be an evolutionary feature that aims to protect these digestion sites (R542, K544 and K554 as discussed earlier) that cannot be naturally mutated due their functional importance.

Another interesting application of our findings could be in the design and development of an oral vaccine against HEV. One of the major obstacles in oral vaccination is the notoriously weak- or non-immunogenicity of vaccines once ingested, which is mainly caused by proteolysis^27^. For the proteins we investigated here, the trypsinization did not affect their antigenicity and therefore could be regarded as potential oral vaccine candidates especially the p146 that was not affected by the trypsin treatment. However, further experimental designs are needed to investigate in depth this application.

## Methods

### Plasmids, antibodies and reagents

Plasmid pET-28a (+)/p179 containing the 439–617aa region of HEV ORF2 of genotype 4 HEV strain has been constructed previously in our laboratory^14,28^. The p179 mutants with wild-type asparagine (N) replaced by the cyclic aa (P) and aromatic aa (Y) at position 562 were previously designed and successfully expressed in *P. pastoris*^14^. HEV capsid protein-specific mAb (1G10) was produced by our research group^13^. Isopropyl-β-D-thiogalactopyranoside (IPTG), High-fidelity DNA polymerase, dNTP, T4 DNA ligase and restriction endonucleases were purchased from Roche (Germany). *E. coli* BL21(DE3) cells were purchased from Promega. HRP-conjugated goat anti-mouse was from KPL (Gaithersburg, MD, USA). Trypsin and DAB were purchased from Sigma–Aldrich (St. Louis, MO, USA). Plasmid and DNA recovery/purification kits were obtained from Axygen, Inc (USA). The nickel-nitrilotriacetic acid (Ni-NTA) Agarose was obtained from QIAGEN Sciences, MD, USA.

### Plasmid construction

The truncated capsid protein p146, p216, p222 were respectively generated from amino acid positions 460–605, 422–637 and 439–660 of open reading frame 2 (ORF2) of genotype 4 HEV strain H4-NJ703. Protein p146, p216, p222, p179, p179/N562P and p179/N562Y were expressed in *E. coli*. Briefly, the different DNA coding sequences were amplified by polymerase chain reaction (PCR) and modified to contain the endonuclease restriction sites 5′-NcoI and 3′-XhoI using the primers listed in Table 4. The desired PCR products were then purified using QIAprep® Spin Miniprep Kit. Next, purified DNA coding sequences were digested with NcoI and XhoI endonucleases, then inserted into the pET-28a (+) vector using T4 DNA ligase. Recombinant plasmids were transformed into competent *E. coli* BL21(DE3) cells. Gene structures were confirmed by endonuclease digestion and DNA sequencing.

**Table 4:** List of the primers used for the amplification of the different DNA coding sequences.

Primers	sequence (5′ to 3′)
p146 (aa460–605)	F: TTT CCA TGG GCCGCCCTTTTTCTGTGCTT
	R: TTT CTC GAG AGAATGGGGTGCGAGGA
p179 (aa439–617)	F:CCC CCATGG TTATCCAGGACTATGATAATC
	R:CCC CTCGAG GACAGTGTCCTCCAA AAC
p216 (aa422–637)	F: CCC CCATGG ATAAGGGGATAGCTATC
	R: CCC CTCGAG GCCCTGAAGGCCGAGCGC
p222 (aa439–660)	F: CCC CCATGG TTATCCAGGACTATGATAATC
	R: CCC CATGAG AGCAGTAGTATCATAATTGTA

### Expression and purification of recombinant proteins

After screening on LB plates and verifying transformants with genomic PCR, cells harboring complete wild-type and mutant expression cassettes were grown in LB medium at 37 °C until reached an optical density of 0.6 at 600 nm. Then, the expression was induced for a 2–3 h by adding IPTG to a final concentration of 1 mM. After the incubation period with constant shaking, the cells were pelleted and lysed. The proteins were N-terminally His-tagged, thus purified by Ni–NTA affinity chromatography as follows: Cell pellets were suspended in binding buffer (50 mM NaH2PO4, pH 8.0, containing 300 mM NaCl and 1 mM phenylmethylsulfonyl fluoride [PMSF]) and lysed by lysozyme. The suspensions were clarified by centrifugation (14,000 × g for 30 min) and then the supernatants were loaded separately onto columns containing Ni–NTA superflow affinity resin, equilibrated with the binding buffer. The columns were washed with five column volumes of binding buffer containing 10 mM of imidazole, and the fusion proteins were eluted by the same buffer containing 250 mM imidazole as described previously^29^.

### Trypsin digestion assay

The six produced HEV recombinant proteins were digested *in vitro* with trypsin as follows: Ten micoliters of purified proteins (1 mg/ml) were subjected to tryptic digestion using 10 μl of an artificial intestinal juice (KH2PO4 6.8 g/L, trypsin 10.0 g/L, pH = 7.5) for 120 min at 37 °C, since this temperature is optimal for trypsin activity and also close to body temperature.

### SDS-polyacrylamide gel electrophoresis and Western blot analysis

After incubation for 120 min, 20 μl aliquots of samples were mixed with 4 μl 6× SDS-PAGE loading buffer [300 mM Tris-cl pH 6.0, 12% (m/v) SDS, 12% (m/v) Bromophenol blue, 60% (v/v) glycerol, 600 mM β-mercaptoethanol], vortexed for 1 min and boiled for 5 min and then were electrophoresed on 15% SDS-polyacrylamide gel (20 μl loaded per lane). For the non-reducing SDS gel, the buffer contained only 0.1% SDS, no β-mercaptoethanol, and the sample was not boiled^8^.

The proteins were heated in 2× loading buffer and electrophoresed in a 15% SDS-PAGE. Electrophoretic transfer of each protein to a nitrocellulose membrane was carried out at 200 mA for 90 min at 4 °C. After transfer, the membrane was immersed for 2 h in a blocking solution (5% skim milk in TBST) and washed with TBST. The membrane was incubated overnight at 4 °C with 1:200 dilution of HEV 1G10 neutralizing mAb^13^ and then washed three times with TBST. A horseradish peroxidase-conjugated anti-mouse IgG was used as the secondary antibody (1:2000 in 5% skim milk in TBST). After 2 h of incubation, the blots were washed and 3,3′- diaminobenzidine (DAB) was added to visualize.

### Proteins 3D structures prediction

The tertiary structures of the P146, P179, P216 and P222, P179/N562P and P179/N562Y were predicted using Phyre2 server^30^. The results were refined using GalaxyWeb^31^. To evaluate the quality of the predicated 3D structures, Molprobity^32^ was used and the best models were selected for further analysis. The P146, P179, P216 and P222 dimers were predicted and assessed as previously described^14^.

### Prediction of interface residues via protein-docking

ZDOCK server was used for protein-protein docking where the crystal structure of bovine trypsin (PDB ID code: 418 G) retrieved from the protein data bank was set as ligand^33^; and the predicted 3D structural models of HEV ORF2 proteins were set as the receptor. Docking results were optimized using the RDOCK algorithm^34^. The results obtained from the ZDOCK were further refined and re-ranked to select docking poses that theoretically occur spontaneously in a solvent environment. All structures were observed and analyzed using python based PYMOL molecular graphics system, Version 1.8 Schrödinger, LLC. Proteolytic cleavage sites were selected according to the relative position of trypsin cleavage site and trypsin catalytic triad H57-D102-S195^17^. Docking solutions were abandoned when trypsin cleavage site was far away from the catalytic triad.

### Analysis of trypsin cleavage sites

Two different methods were used to analyze the exposure of all of the trypsin cleavage sites (K/R residues) in the different HEV proteins. First, the solvent accessibilities of the selected K/R residues were calculated using Discovery Studio 3.0 (Accelrys Inc., San Diego, USA). The residue solvent accessibility area (RSA) is the sum of the surface of all atoms, including the backbone atoms; the percent solvent accessibility (PSA) is 100 times the RSA divided by the residue solvent accessibility of the fully exposed amino acid residue calculated using the extended Ala-X-Ala tripeptide, where X is the residue of interest^35,36^. Second, the protrusion index (PI) of trypsin cleavage sites was also calculated using ElliPro server^37^. In Ellipro, the 3D structure of the protein is approximated by a number of ellipsoids, that the ellipsoid with PI = 0.9 would include within 90% of the protein residues with 10% of the protein residues being outside of the ellipsoid; while the ellipsoid with PI = 0.8 would include 80% of residues with 20% being outside the ellipsoid. This implicates that the more the PI value is high the more the residue is exposed.

### Conservation of the trypsin proteolytic sites within the HEV ORF2 protein

We retrieved from GenBank the full genomes of the 137 HEV strains that were used by Smith *et al*. for the phylogenetic analysis and classification of the family Hepeviridae. The accession numbers are listed in supplementary materials. We focused our analysis only on the ORF2 proteins. For the multiple alignment of protein sequences, we used COBALT^38^ included in the NCBI C +  + toolkit and also available at https://www.st-va.ncbi.nlm.nih.gov/tools/cobalt/re_cobalt.cgi. Then the results were visualized and refined manually using Jalview2 program^39^.

### Equipment and settings

The gels and blots are photographed by full automatic gel imaging analysis system. (JS-780, PeiQing Technology Co., Ltd. Shanghai, China). In Figs 1–4, pictures were gathered using PhotoFiltre Studio X 10.12.0 (2001–2017, Antonio Da Cruz).

### Data Availability

The datasets generated and/or analyzed during the current study are available from the corresponding author on reasonable request.

## Electronic supplementary material

Supplementary materials
