# Author Correction: Dimerization: a structural feature for the protection of hepatitis E virus capsid protein against trypsinization

**DOI:** 10.1038/s41598-018-24852-8

**Published:** 2018-04-30

**Authors:** Wenjuan Wei, Nouredine Behloul, Sarra Baha, Zhenzhen Liu, Mehwish Saba Aslam, Jihong Meng

**Affiliations:** Department of Microbiology and Immunology, School of Medicine, Southeast University, Nanjing, Jiangsu province China

Correction to: *Scientific Reports* 10.1038/s41598-018-20137-2, published online 29 January 2018

This Article contains errors in References 8 and 9 which are incorrectly given as:

‘Yau, W. *et al*. Colistin hetero-resistance in multidrug-resistant Acinetobacter baumannii clinical isolates from the Western Pacific region in the SENTRY antimicrobial surveillance programme. *J Infection* **58**, 138–144, 10.1016/j.jinf.2008.11.002 (2009).’

‘Rahim, N. A. *et al*. Synergistic killing of NDM-producing MDR Klebsiella pneumoniae by two ‘old’ antibiotics-polymyxin B and chloramphenicol. *J Antimicrob Chemoth* **70**, 2589–2597, 10.1093/jac/dkv135 (2015).’

The correct references are listed below as refs^1,2^.

## References

[1] Li S (2009). Dimerization of hepatitis E virus capsid protein E2s domain is essential for virus-host interaction. Plos Pathog.

[2] Li S, Zhang J, Xia N (2015). Lessons from hepatitis E vaccine design. Curr Opin Virol.

