# Supplementary material for: Dimerization: a structural feature for the protection of hepatitis E virus capsid protein against trypsinization
Source: Sci Rep. 2018 Jan 29;8:1738. doi: 10.1038/s41598-018-20137-2 (PMC5788867; doi:10.1038/s41598-018-20137-2)
Supplement: Supplementary file 1 — Supplementary materials [file 41598_2018_20137_MOESM1_ESM.pdf]

Title: Dimerization: a structural feature for the protection of hepatitis E virus capsid protein against trypsinization

Author list: Wenjuan Wei, Nouredine Behloul, Sarra Baha, Zhenzhen Liu, Mehwish Saba Aslam and Jihong Meng

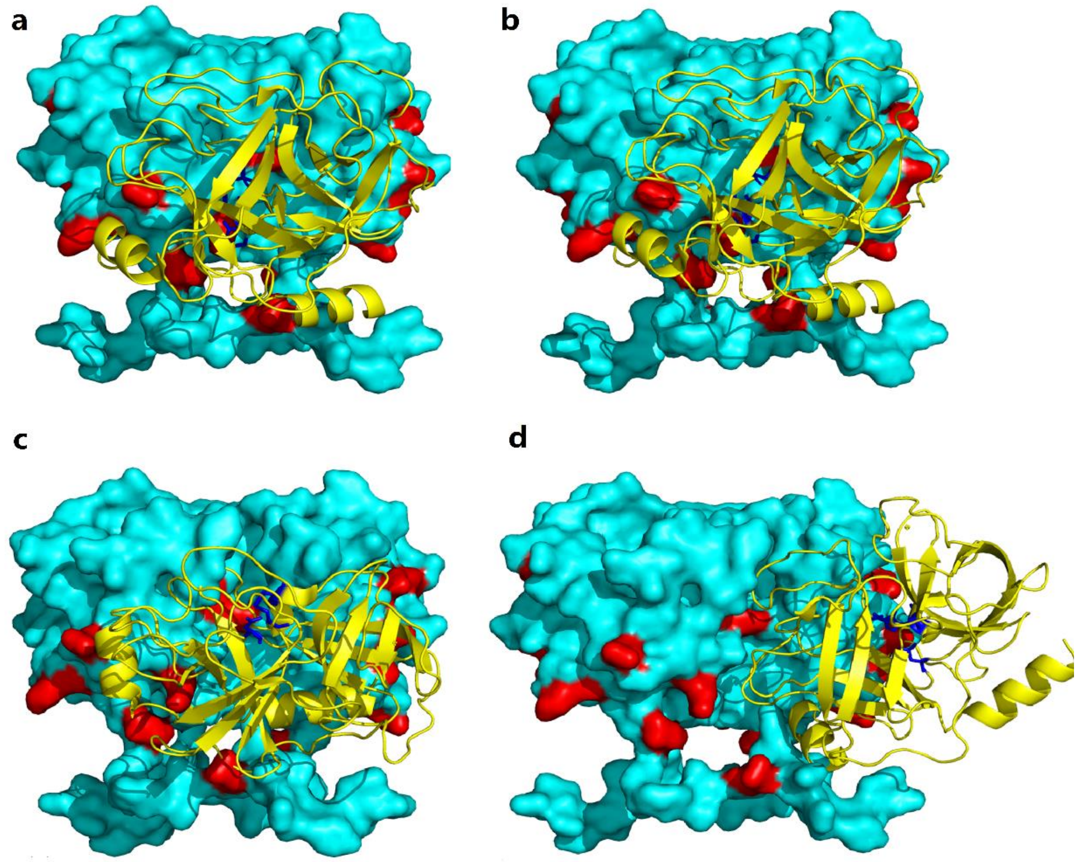

Figure S1. The best docking poses of the p179 homodimer and trypsin (PDB ID: 418G). The p179 dimer is shown in cyan surface representation with the different trypsin digestion sites depicted in red. Trypsin is show in yellow cartoon representation with its catalytic triad rendered in blue sticks.

	K423	R437	R451	R460	R466	R512	K518	R524	K536	R542	R544	K554	R578	R619	R631	R649	K651	K653	K656	R658
K423	25.91	24.44	22.85	21.96	21.11	16.17	15.46	14.97	13.66	<13	<13	<13	<13	<13	<13	<13	<13	<13	<13	<13
R437	24.44	24.44	22.85	21.96	21.11	16.17	15.46	14.97	13.66	<13	<13	<13	<13	<13	<13	<13	<13	<13	<13	<13
R451	22.85	22.85	22.85	21.96	21.11	16.17	15.46	14.97	13.66	<13	<13	<13	<13	<13	<13	<13	<13	<13	<13	<13
R460	21.96	21.96	21.96	21.96	21.11	16.17	15.46	14.97	13.66	<13	<13	<13	<13	<13	<13	<13	<13	<13	<13	<13
R466	21.11	21.11	21.11	21.11	21.11	16.17	15.46	14.97	13.66	<13	<13	<13	<13	<13	<13	<13	<13	<13	<13	<13
R512	16.17	16.17	16.17	16.17	16.17	16.17	15.46	14.97	13.66	<13	<13	<13	<13	<13	<13	<13	<13	<13	<13	<13
K518	15.46	15.46	15.46	15.46	15.46	15.46	15.46	14.97	13.66	<13	<13	<13	<13	<13	<13	<13	<13	<13	<13	<13
R524	14.97	14.97	14.97	14.97	14.97	14.97	14.97	14.97	13.66	<13	<13	<13	<13	<13	<13	<13	<13	<13	<13	<13
K536	13.66	13.66	13.66	13.66	13.66	13.66	13.66	13.66	13.66	<13	<13	<13	<13	<13	<13	<13	<13	<13	<13	<13
R542	<13	<13	<13	<13	<13	<13	<13	<13	<13	13.46	13.46	13.46	13.46	13.46	13.46	13.46	13.46	13.46	13.46	13.46
R544	<13	<13	<13	<13	<13	<13	<13	<13	<13	13.46	13.65	13.65	13.65	13.65	13.65	13.65	13.65	13.65	13.65	13.65
K554	<13	<13	<13	<13	<13	<13	<13	<13	<13	13.46	13.65	14.77	14.77	14.77	14.77	14.77	14.77	14.77	14.77	14.77
R578	<13	<13	<13	<13	<13	<13	<13	<13	<13	13.46	13.65	14.77	17.38	17.38	17.38	17.38	17.38	17.38	17.38	17.38
R619	<13	<13	<13	<13	<13	<13	<13	<13	<13	13.46	13.65	14.77	17.38	21.51	21.51	21.51	21.51	21.51	21.51	21.51
R631	<13	<13	<13	<13	<13	<13	<13	<13	<13	13.46	13.65	14.77	17.38	21.51	22.96	22.96	22.96	22.96	22.96	22.96
R649	<13	<13	<13	<13	<13	<13	<13	<13	<13	13.46	13.65	14.77	17.38	21.51	22.96	24.86	24.86	24.86	24.86	24.86
K651	<13	<13	<13	<13	<13	<13	<13	<13	<13	13.46	13.65	14.77	17.38	21.51	22.96	24.86	25.10	25.10	25.10	25.10
K653	<13	<13	<13	<13	<13	<13	<13	<13	<13	13.46	13.65	14.77	17.38	21.51	22.96	24.86	25.10	25.36	25.36	25.36
K656	<13	<13	<13	<13	<13	<13	<13	<13	<13	13.46	13.65	14.77	17.38	21.51	22.96	24.86	25.10	25.36	25.60	25.60
R658	<13	<13	<13	<13	<13	<13	<13	<13	<13	13.46	13.65	14.77	17.38	21.51	22.96	24.86	25.10	25.36	25.60	25.86

K423 R437 R451 R460 R466 R512 K518 R524 K536 R542 R544 K554 R578 R619 R631 R649 K651 K653 K656 R658

a

	K423	R437	R451	R460	R466	R512	K518	R524	K536	R542	R544	K554	R578	R619	R631	R649	K651	K653	K656	R658
K423	<13	<13	<13	<13	<13	<13	<13	<13	<13	13.27	13.45	14.57	17.2	21.3	22.75	24.62	24.86	25.12	25.36	25.62
R437	<13	<13	<13	<13	<13	<13	<13	<13	<13	<13	<13	13.1	15.72	19.85	21.27	23.15	23.39	23.65	23.89	24.15
R451	<13	<13	<13	<13	<13	<13	<13	<13	<13	<13	<13	<13	14.13	18.26	19.69	21.56	21.8	22.06	22.3	22.56
R460	<13	<13	<13	<13	<13	<13	<13	<13	<13	<13	<13	<13	13.24	17.37	18.79	20.67	20.91	21.17	21.41	21.67
R466	<13	<13	<13	<13	<13	<13	<13	<13	<13	<13	<13	<13	<13	16.52	17.94	19.81	20.05	20.31	21.56	20.81
R512	<13	<13	<13	<13	<13	<13	<13	<13	<13	<13	<13	<13	<13	<13	13.01	14.88	15.12	15.38	15.63	15.88
K518	<13	<13	<13	<13	<13	<13	<13	<13	<13	<13	<13	<13	<13	<13	<13	14.17	14.41	14.67	14.91	15.17
R524	<13	<13	<13	<13	<13	<13	<13	<13	<13	<13	<13	<13	<13	<13	<13	13.68	13.92	14.18	14.42	14.68
K536	<13	<13	<13	<13	<13	<13	<13	<13	<13	<13	<13	<13	<13	<13	<13	<13	<13	<13	13.11	13.36
R542	13.27	<13	<13	<13	<13	<13	<13	<13	<13	<13	<13	<13	<13	<13	<13	<13	<13	<13	<13	<13
R544	13.45	<13	<13	<13	<13	<13	<13	<13	<13	<13	<13	<13	<13	<13	<13	<13	<13	<13	<13	<13
K554	14.57	13.1	<13	<13	<13	<13	<13	<13	<13	<13	<13	<13	<13	<13	<13	<13	<13	<13	<13	<13
R578	17.2	15.72	14.13	13.24	<13	<13	<13	<13	<13	<13	<13	<13	<13	<13	<13	<13	<13	<13	<13	<13
R619	21.3	19.85	18.26	17.37	16.52	<13	<13	<13	<13	<13	<13	<13	<13	<13	<13	<13	<13	<13	<13	<13
R631	22.75	21.27	19.69	18.79	17.94	13.01	<13	<13	<13	<13	<13	<13	<13	<13	<13	<13	<13	<13	<13	<13
R649	24.62	23.15	21.56	20.67	19.81	14.88	14.17	13.68	<13	<13	<13	<13	<13	<13	<13	<13	<13	<13	<13	<13
K651	24.86	23.39	21.8	20.91	20.05	15.12	14.41	13.92	<13	<13	<13	<13	<13	<13	<13	<13	<13	<13	<13	<13
K653	25.12	23.65	22.06	21.17	20.31	15.38	14.67	14.18	12.86	<13	<13	<13	<13	<13	<13	<13	<13	<13	<13	<13
K656	25.36	23.89	22.3	21.41	21.56	15.63	14.91	14.42	13.11	<13	<13	<13	<13	<13	<13	<13	<13	<13	<13	<13
R658	25.62	24.15	22.56	21.67	20.81	15.88	15.17	14.68	13.36	<13	<13	<13	<13	<13	<13	<13	<13	<13	<13	<13

K423 R437 R451 R460 R466 R512 K518 R524 K536 R542 R544 K554 R578 R619 R631 R649 K651 K653 K656 R658

b

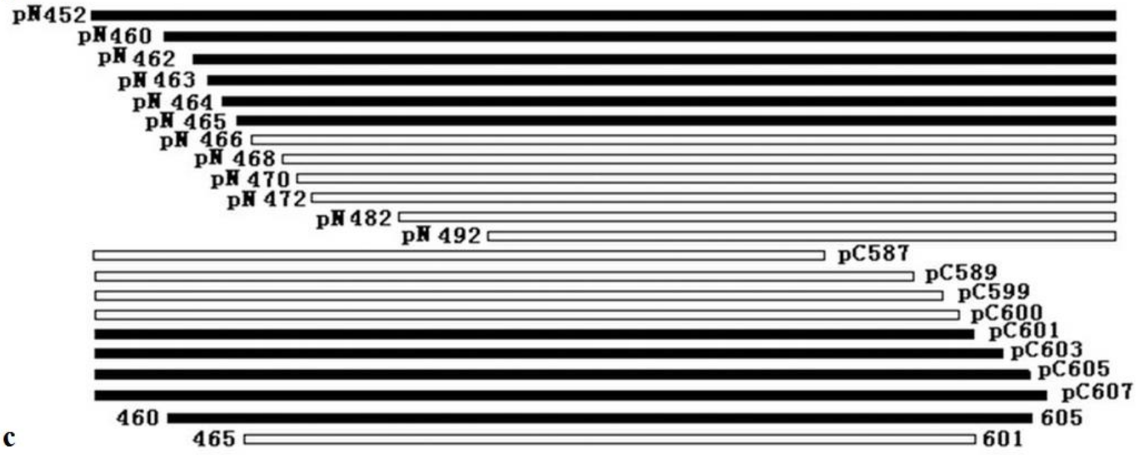

c

Figure S2. Summary of all the possible products after the trypsinization of the region aa 422-660 of the HEV ORF2 protein and the reactivity of different truncated ORF2 proteins against HEV neutralizing antibody 1G10. (a) The upper left triangle shows the molecular weight of the C-terminal trypsinization product if the cleavage occurred in the N-terminal region and this latter was fully degraded. The upper left triangle shows the molecular weight of the C-terminal trypsinization product if the cleavage occurred in the N-terminal region and this latter was fully degraded. The bottom right triangle shows the molecular weight of the N-terminal trypsinization product if the cleavage occurred in the C-terminal region and this latter was fully degraded. Part (b) shows molecular weight of the middle segment if a trypsin double cleavage occurred: on in the N-terminal region and the other in the C-terminal region and both N- and C-terminal resulting fragments were fully degraded. In both (a) and (b), the red highlight indicates that the resulting fragment cannot react against the 1G10 monoclonal antibody whereas the green highlight indicates a positive reaction. The dark-green highlight indicates that the molecular weight of the fragment corresponds to the bands observed in the Western blot analyses (17-18 kDa). (c) Schematic of truncated HEV ORF2 proteins and their reactivities with mAb 1G10[13]. White bars represent the truncated proteins that had no detectable reactivity with the neutralizing mAb 1G10, while the black ones indicated that they had strong reactivity with 1G10. The length of the bars indicated the region covered by the truncated proteins.

Figure S3.

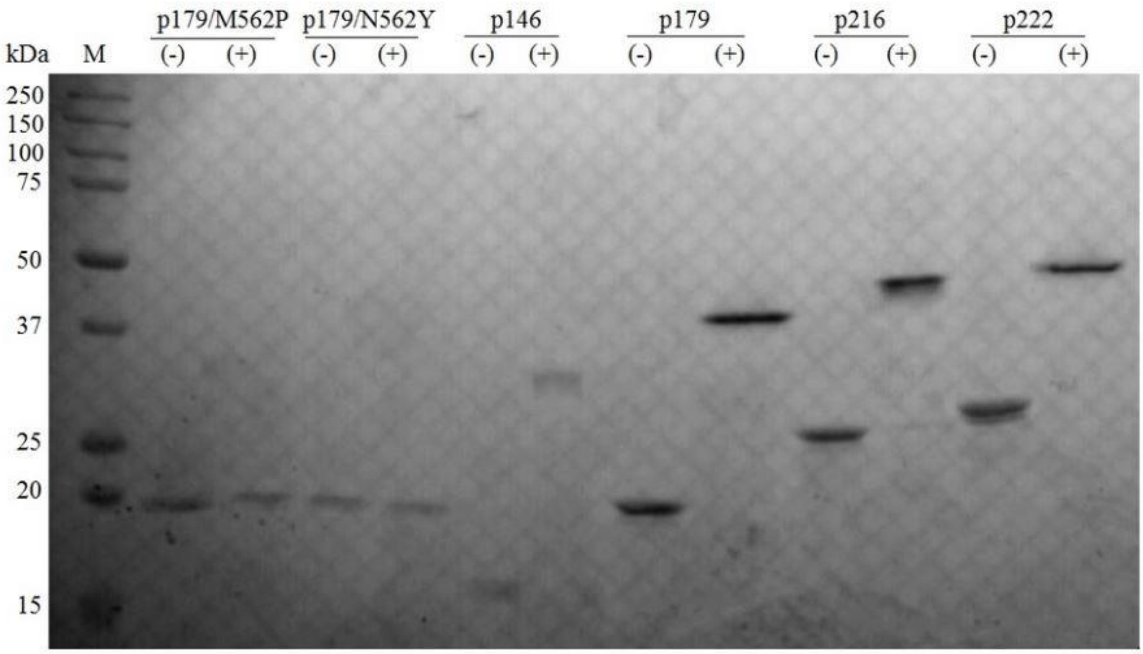
p179/M562P
p179/N562Y
p146
p179
p216
p222
kDa
M
(-)
(+)
250
150
100
75
50
37
25
20
15

Figure S4.

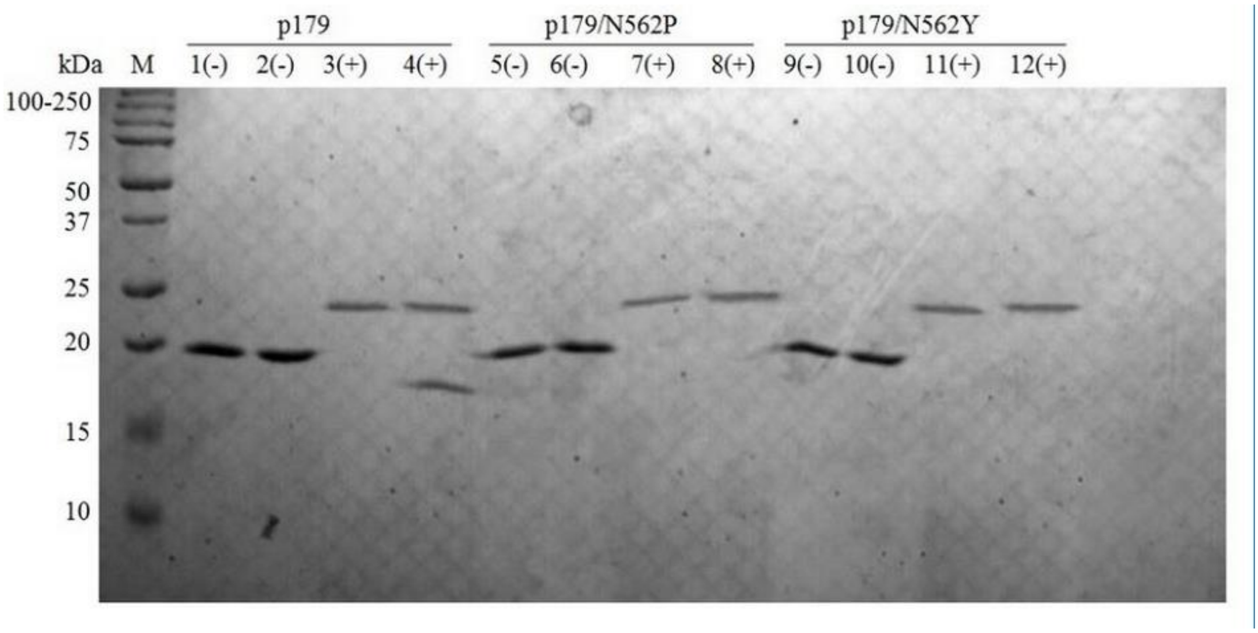

p179
p179/N562P
p179/N562Y
kDa
M
1(-)
2(-)
3(+)
4(+)
5(-)
6(-)
7(+)
8(+)
9(-)
10(-)
11(+)
12(+)
100-250
75
50
37
25
20
15
10

Figure S5.

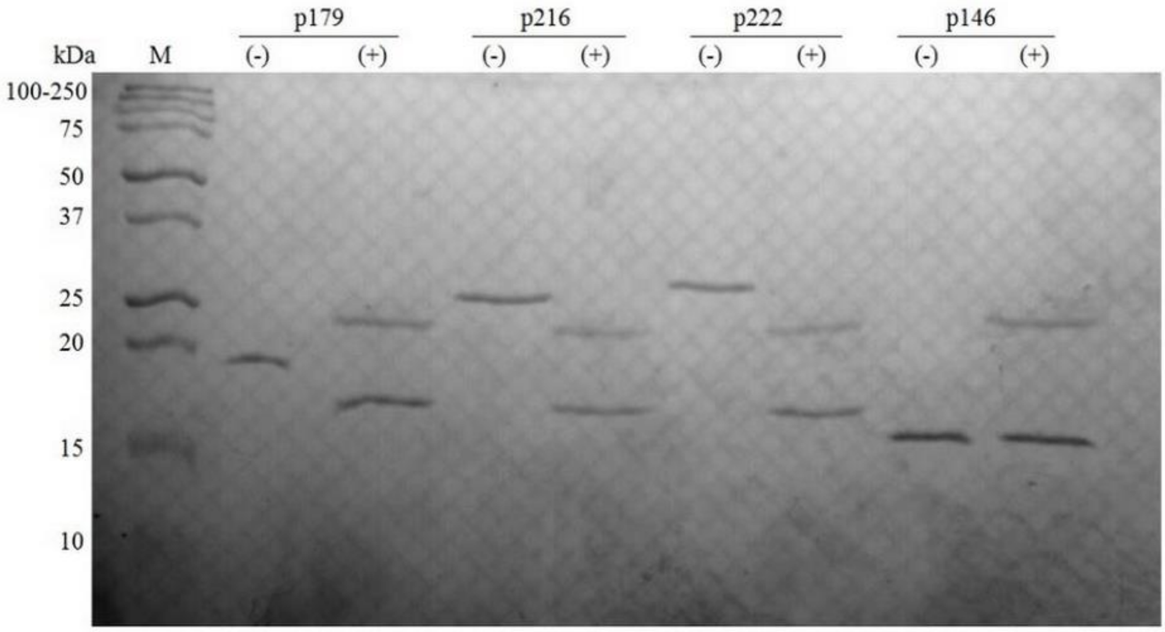

Figure S6.

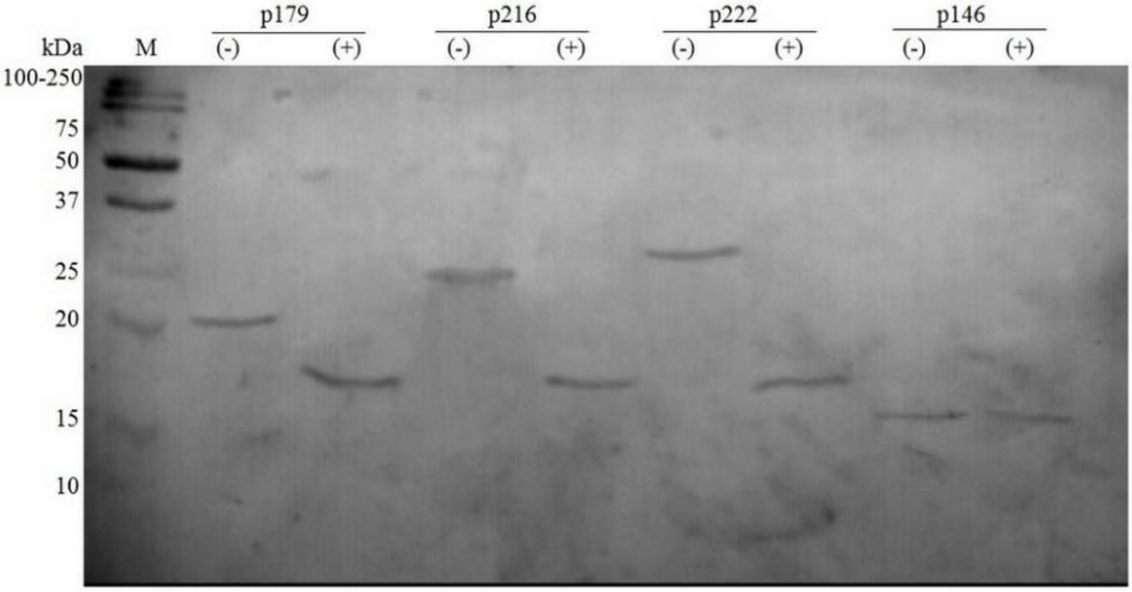

Table S1. Conservation of the 20 K/R sites among 137 HEV strains

**Proteolytic sites**	**Conservation rate**	**No. of mutated sequences**	**Mutations**	**Genotype, country**	**Accession number of the mutated sequences**
**K423**	100%	0			
**R437**	100%	0			
R451	99.3%	1	RΔQ	Rabbit, China,	FJ906895
**R460**	100%	0			
**R466**	100%	0			
**R512**	100%	0			
K518	98.5%	2	KΔR	4, Japan<br>1, India	AB253420<br>JF443722
R524	98.5%	2	RΔQ	Camel, United Arab Emirates,	KJ496143<br>KJ496144
K536	99.3%	1	KΔR	Wild boar, Japan	AB856243
**R542**	100%	0			
**R544**	100%	0			
K554	97.8%	3	KΔR	1, India<br>3, USA<br>3, UK	AF076239<br>HQ709170<br>HQ389543
**R578**	100%	0			
R619	99.3%	1	RΔC	3, South Korea	FJ426403
**R631**	100%	0			
R649	99.3%	1	RΔP	3, China	FJ527832
K651	98.5%	2	KΔR	3, Spain	EU723514<br>EU723516
K653	98.5%	2	KΔE	3, USA<br>Wild boar, Japan	HQ709170<br>AB573435
**K656**	100%	0			
**R658**	100%	0			
